# Role of Autophagy in Type 2 Diabetes Mellitus: The Metabolic Clash

**DOI:** 10.1111/jcmm.70240

**Published:** 2024-12-10

**Authors:** Yousef Abud Alanazi, Haydar M. Al‐kuraishy, Ali I. Al‐Gareeb, Athanasios Alexiou, Marios Papadakis, Mostafa M. Bahaa, Walaa A. Negm, Faisal Holil AlAnazi, Mohammed Alrouji, Gaber El‐Saber Batiha

**Affiliations:** ^1^ Department of Pediatrics, College of Medicine Majmaah University Majmaah Saudi Arabia; ^2^ Department of Clinical Pharmacology and Medicine, College of Medicine Mustansiriyah University Baghdad Iraq; ^3^ University Centre for Research & Development Chandigarh University Mohali Punjab India; ^4^ Department of Research & Development Funogen Athens Greece; ^5^ Department of Surgery II University Hospital Witten‐Herdecke, University of Witten‐Herdecke Wuppertal Germany; ^6^ Pharmacy Practice Department, Faculty of Pharmacy Horus University New Damietta Egypt; ^7^ Department of Pharmacognosy, Faculty of Pharmacy Tanta University Tanta Egypt; ^8^ Department of Internal Medicine, College of Medicine Majmaah University Majmaah Saudi Arabia; ^9^ Department of Clinical Laboratory Sciences, College of Applied Medical Sciences Shaqra University Shaqra Saudi Arabia; ^10^ Department of Pharmacology and Therapeutics, Faculty of Veterinary Medicine Damanhour University Damanhour AlBeheira Egypt

**Keywords:** autophagy, pancreatic β cell, type 2 diabetes mellitus

## Abstract

Type 2 diabetes mellitus (T2DM) is developed due to the development of insulin resistance (IR) and pancreatic β cell dysfunction with subsequent hyperglycaemia. Hyperglycaemia‐induced oxidative stress and endoplasmic reticulum (ER) stress enhances inflammatory disorders, leading to further pancreatic β cell dysfunction. These changes trigger autophagy activation, which recycles cytoplasmic components and injured organelles. Autophagy regulates pancreatic β cell functions by different mechanisms. Though the exact role of autophagy in T2DM is not completely elucidated, that could be beneficial or detrimental. Therefore, this review aims to discuss the exact role of autophagy in the pathogenesis of T2DM.

## 1 Introduction

Type 2 diabetes mellitus (T2DM) is a long‐lasting metabolic disorder due to the development of insulin resistance (IR) and subsequent hyperglycaemia [1]. T2DM is associated with low‐grade inflammatory disorders due to hyperglycaemia‐mediated oxidative stress and the release of pro‐inflammatory cytokines [2]. IR and relative insulin deficiency caused by pancreatic β cell dysfunction are the chief pathology of T2DM [3]. Of note, the initiation of inflammatory disorders in T2DM is developed due to the deregulation of immune cells, progressive expression of pro‐inflammatory cytokines and progress of systemic inflammation [4]. Notably, chronic low‐grade inflammation in T2DM triggered by hyperglycaemia and adipose tissue activation raises the progression of IR and related complications [5]. In addition, inflammatory disorders contribute to the development of IR, T2DM and systemic complications [6].

**FIGURE 1:**
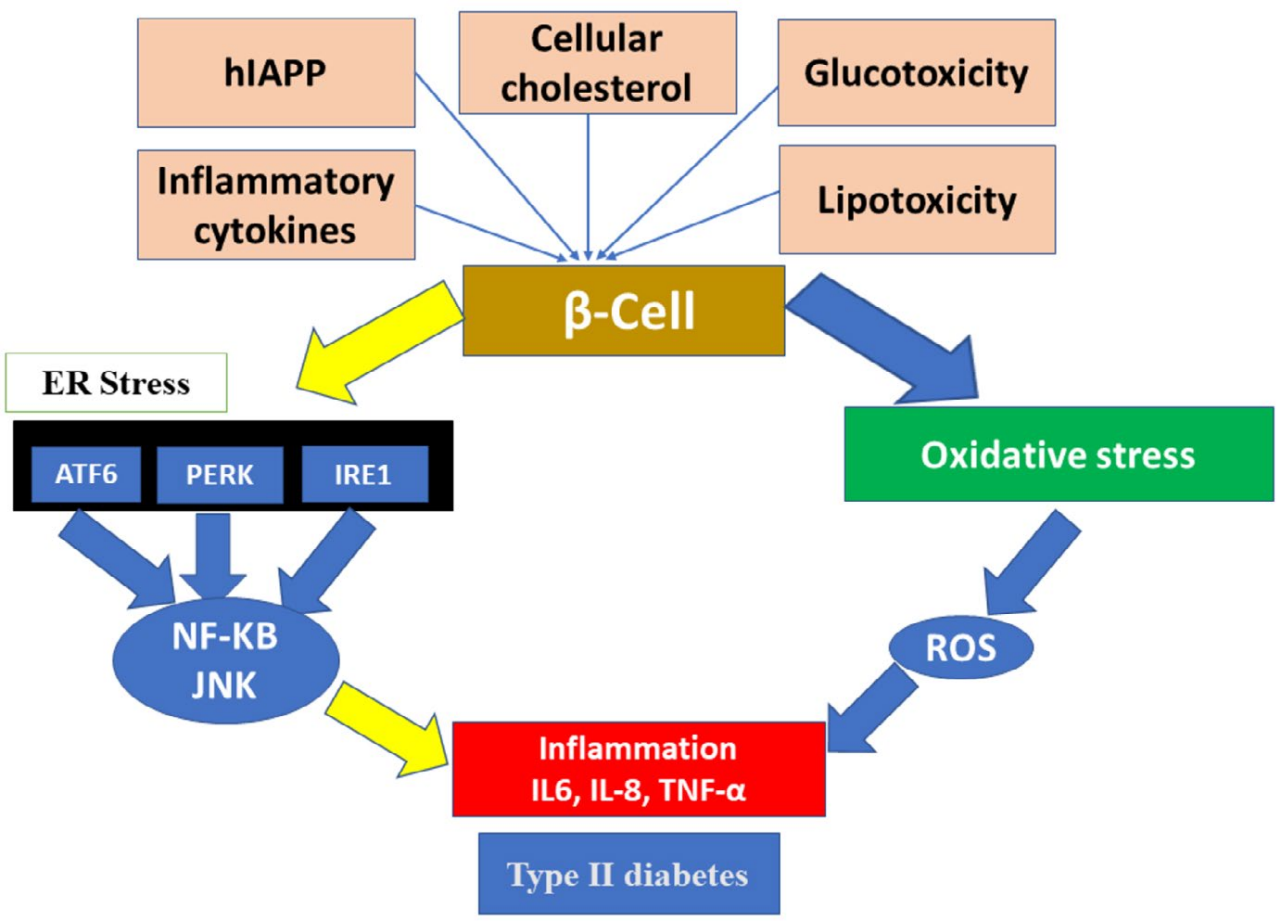
Inflammatory disorders and development of T2DM.

Furthermore, hypoglycaemia, hyperglycaemia and glucose variability stimulate oxidative stress, enhancing inflammatory disorders [7]. Also, environmental and genetic factors such as stress, diet and smoking are intricated with the initiation of chronic inflammation in T2DM [8]. Furthermore, abnormal deposition of cellular cholesterol, human amyloid polypeptide, lipotoxicity, glucotoxicity and inflammatory cytokines contribute to developing pancreatic β cell dysfunction [9]. Further, pancreatic β cell dysfunction induces the progression of oxidative stress and endoplasmic reticulum (ER) stress which, via activation of inflammatory signalling pathways, promote inflammatory disorders and the development of T2DM [10] (Figure 1).

These inflammatory changes trigger the activation of different cellular processes as compensatory mechanisms to decrease IR and adipose tissue dysfunction in T2DM [11]. One important cellular process engaged with the recycling of cytoplasmic components and injured organelles is autophagy which is triggered in response to cellular stress, as in oxidative stress and inflammatory reactions [12]. It has been shown that reactive nitrogen species (RNS) and reactive oxygen species (ROS) play an important role in the sustained activation of autophagy [13]. In turn, autophagy regulates pancreatic β cell functions by different mechanisms [14]. It has been shown that dysregulated autophagy is implicated in the pathogenesis of IR though, suppression of autophagy promotes pancreatic β cell deaths [15]. Therefore, pancreatic β cell autophagy seems as an adaptive response to alleviate the development of IR. As well, activation of autophagy reduces apoptosis of pancreatic β cells induced by lipotoxicity [16]. On the other hand, excessive autophagy activation is linked with the induction of autophagic cell death and apoptosis of pancreatic β cells [17]. The role of autophagy in T2DM may be a double sword edge [18]. However, the precise role of autophagy in T2DM is not fully elucidated. Therefore, this review aims to discuss the exact role of autophagy in T2DM.

## 2 Autophagy Overview

The word autophagy was coined from the Greek word, which means self‐eating. In the 1960s, autophagy was observed as a process to form vesicles that are transported to the lysosomes for degradation [19, 20]. Later, autophagy word was often used in miscellaneous articles in the middle of the 19th century [19]. Christian de Duve, a Belgian biochemist, was the first who designate autophagy in 1963 [20]. In the 1990s numerous researchers presume several mechanisms of autophagy [21, 22]. Yoshinori Ohsumi, in 2016 awarded a Nobel Prize in physiology for the discovery of autophagy [23].

Autophagy is an essential degradation process to abolish abnormal cellular components within the cells [24]. Three chief autophagy types are documented: micro‐autophagy, macro‐autophagy and chaperon‐mediated autophagy (CMA) [25]. In macro‐autophagy, the phagophore surrounds the agent's requisites to be engulfed and form autophagosomes. In the lysosome, the autophagosome contents are degraded by acidic lysosomal hydrolase [26]. However, cytoplasmic contents are consumed in micro‐autophagy and directed towards the lysosomes [27].

The foremost type of autophagy is macro‐autophagy, subdivided into selective autophagy, which eradicates aggregated proteins [28], and non‐selective autophagy, which provides nutrients to the cells during starvation [29]. Selective autophagy encourages intracellular homeostasis by eliminating aggregated proteins, injured organelles and pathogens [30]. Furthermore, the requisitioning of cargo in the autophagosomes is required for the molecular apparatus of selective autophagy. Cargo proteins drag cargo receptors on the autophagosome membrane. For example, selective autophagy of mitochondria is called mitophagy [30]. Non‐selective autophagy enhances the eradication of injured organelles through the dysregulation of nutritional and environmental conditions in response to starvation [31]. CMA is a precise and complex pathway that identifies only heat shock protein 70‐containing proteins and promotes the establishment of the CMA–protein complex. This complex binds CMA receptors on the lysosomal membrane, and the protein contents are translocated across lysosomal membranes and degraded by specific enzymes [32]. CMA is intricate in degrading specific proteins, DNA repair and cellular reprogramming in reaction to diverse stressful factors [33]. CMA is included in translocating unfolded proteins into the lysosomes for degradation by binding chaperone proteins with diverse substrates for lysosomal clearance [33, 34]. CMA failure is associated with cancer and neurodegeneration development [33]. Furthermore, ferroptosis is observed as a specific type of autophagy due to the formation of autophagosomes in response to ferroptosis activators which contribute to ferroptotic cell deaths [34].

**FIGURE 2:**
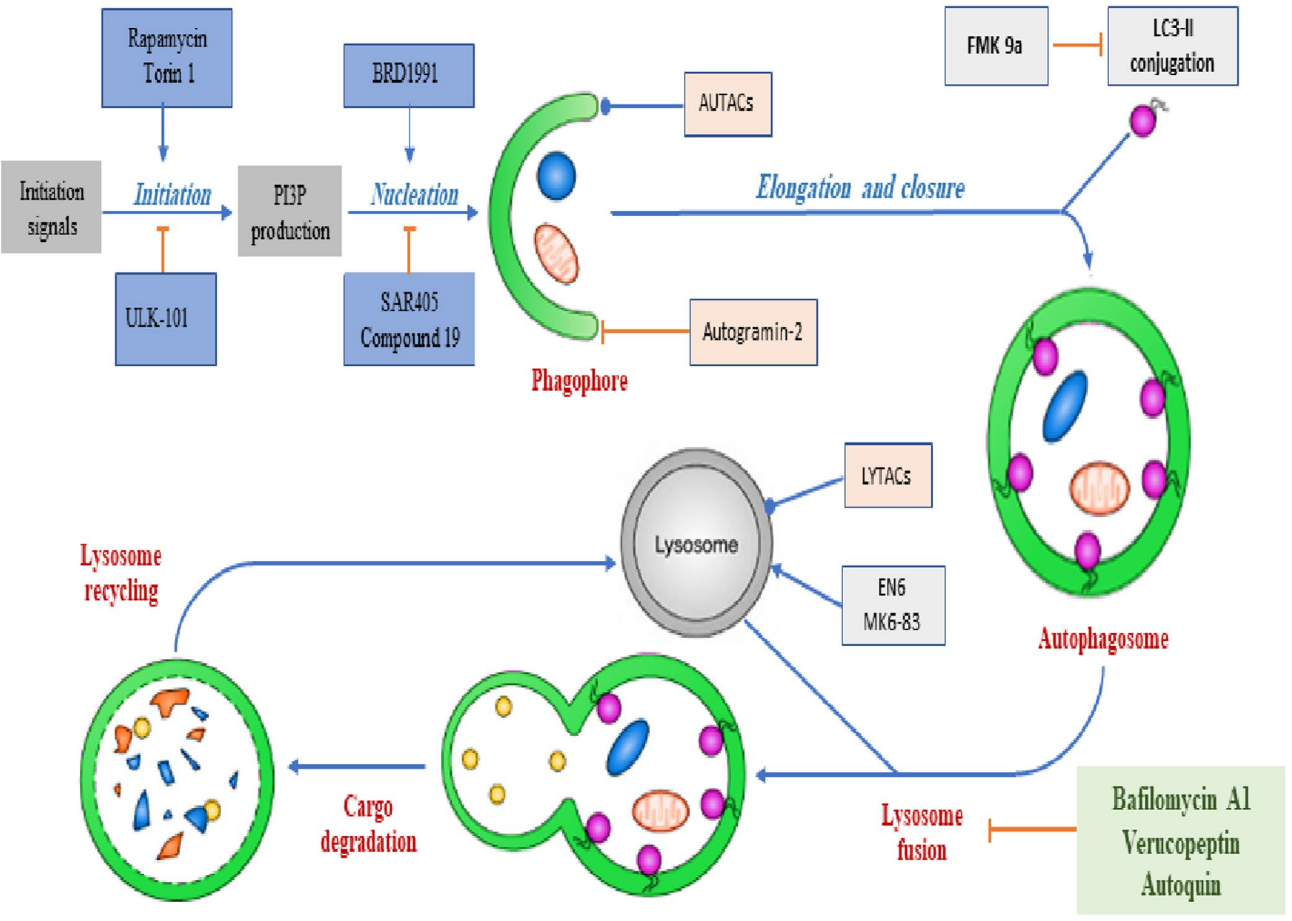
Molecular pathway of autophagy.

Autophagy is organised by autophagy‐related genes (Atg) [35]. Additionally, autophagy function is controlled by adenosine monophosphate kinase (AMPK) and kinases mammalian target of rapamycin (mTOR) through phosphorylation of autophagy‐activated kinases ULK1 and ULK2 [36]. Phosphorylated ULK activates the expression of Beclin‐1, which is a homologue of Atg6 [37]. ULK‐Beclin‐1 complex increases the formation of autophagosomes [38]. Further, surface lipid phosphatidylinositol produces phosphatidylinositol 3 phosphates (PI3P) on the phagophore membrane, which acts as a docking site protein binding [39]. As well, sirtuin‐1 (SIRT1) activates autophagy by inhibiting protein acetylation [40]. SIRT1 regulates the signalling of autophagy, mostly p53 and Foxo3 protein [40]. Consequently, SIRT1‐mediated life extension could be through autophagy activation [40]. The initiation of autophagy signalling promotes the release of PI3P, which is activated by rapamycin and Torin 1 and inhibited by ULK‐101 [40]. PI3P persuades the nucleation process by activating many signalling leads to autophagosome formation [41]. Fusion of autophagosome with lysosome is inhibited by bafilomycin A1, verucopeptin and autoquin. Degradation of cargo protein promotes fusion and interaction of autophagosome with the lysosomes with final degradation contents of autophagosome [42] (Figure 2).

Moreover, autophagy is triggered by starvation by Atg7 signalling when unnecessary proteins are degraded to amino acids, which are recycled to synthesise essential proteins [36]. Dysregulation of the Atg7 gene induces impairment of starvation‐induced autophagy [36]. Also, autophagy is concerned with numerous viral infections. For example, vesicular stomatitis virus is taken up by autophagosome and trans‐located to the endosomes, where it is detected by toll‐like receptor 7 and triggers interferon response [43]. However, some viruses and bacteria can avoid autophagy and use it to replicate [43]. Important, Galectin‐8 is observed as a dangerous receptor that initiates the autophagy pathway against intracellular pathogens [44]. Galectin‐8 can stimulate autophagy adaptor NDP52 and the formation of autophagosomes [44]. Furthermore, Galectin‐8 regulates the interaction between autophagy and lysosomes during viral infections [45]. In addition, autophagy is involved in regulating programmed cell death (PCD) [46]. PCD is an intricate process regulating immune response and neuron functions [46]. Autophagy PCD promotes cell survival and prevents disease progression in different neurodegenerative disorders [47].

Basal autophagy is protective in most tissues by maintaining the homeostasis of cytosolic components. However, induced autophagy in response to starvation, dyslipidemia, infection, and gluco‐lipotoxicity regulates and maintains cellular functions [48]. Induced autophagy is necessary for cell death, called autophagic cell death when apoptosis is inhibited [48]. These findings indicated that autophagy which is regulated by many stimuli is involved in a wide range of diseases. However, the role of autophagy in T2DM may be protective or harmful depending on the underlying inflammation and oxidative.

## 3 Autophagy and T2DM

### 3.1 Protective Effects

Basal autophagy is protective in most tissues by maintaining the homeostasis of cytosolic components. However, induced autophagy in response to starvation, dyslipidemia, infection and gluco‐lipotoxicity regulates and maintains cellular functions [48]. Induced autophagy is necessary for cell death, called autophagic cell death when apoptosis is inhibited [48].

It has been shown that dysregulated autophagy is intricated in the pathogenesis of IR as exposure of pancreatic β cells to long‐chain free fatty acids (FFA) induces autophagy and IR. However, suppression of autophagy enhances FFA‐mediated pancreatic β cell deaths [15]. Thus, pancreatic β cell autophagy seems as an adaptive response to mitigate the development and progression of IR. In vitro study revealed that activation of autophagy attenuates apoptosis of pancreatic β cells induced by lipotoxicity [16]. Different preclinical studies highlighted that over‐activated autophagy could be a compensatory protective mechanism against FFA‐induced apoptosis of pancreatic β cells [15, 49]. Notably, the upregulation of LC3 protects pancreatic β cells from lipotoxicity‐induced apoptosis [16]. As well, defective autophagy promotes the development of diabetes in mice [15]. Autophagy improves pancreatic β cells' function and the release of insulin. Knockout of Atg7 in the pancreatic β cells induces insulin deficiency and impaired glucose homeostasis [50]. Prolong stressful conditions on the pancreatic β cells and accumulation of misfolded proteins trigger the expression of autophagic genes and ER‐associated proteins with subsequent elimination of aggregated proteins [51]. Autophagy preserves the homeostasis of pancreatic β cells and prevents oxidative stress‐induced cellular injury [51]. In Atg‐deficient mice, the number of pancreatic β cells is reduced; abnormal glucose tolerance and reduction of insulin secretion are developed [52]. Lack of autophagy involved in cellular clearance results in the accumulation of hIAPP and the development of overt diabetes in mice [52]. Of note, amyloid‐like or aggregated prone proteins are cleared specifically by autophagy, though clearance of non‐aggregated prone proteins is mainly by proteasomal degradation pathway [17]. Of note, autophagy has a protective role against hIAPP‐induced toxicity of pancreatic β cells [52]. Therefore, induction of the autophagy pathway plays an important role in preventing hIAPP‐induced toxicity of pancreatic β cells.

Deposition of autophagosomes had been observed in mouse diabetic mouse model [17]. Furthermore, activation of autophagy is associated with the reduction of degeneration of pancreatic β cells [49]. Findings from a preclinical study showed that Atg‐deficient mice experienced hypoinsulinemia and hyperglycaemia since; autophagy regulates intracellular content and the release of insulin from pancreatic β cells [53]. In β‐cells of T2DM patients, autophagy is downregulated, the number of autophagic vacuoles and autophagosomes is increased, whereas the expression of lysosomal‐associated membrane protein 2 (LAMP2) and cathepsin is decreased [54]. In addition, lipid droplet accumulation in β‐cells of T2DM patients was accompanied by the inhibition of the translocation of transcription factor EB (TFEB), a master regulator of autophagy to the nucleus and by the down‐regulation of the lysosomal biomarker LAMP2 [55]. Under IR conditions, FFA influx induces autophagy in pancreatic β‐cell death through activation of JNK1, independent of oxidative and ER stress [56]. Atg‐deficient mice revealed hypoinsulinemia and hyperglycaemia, as autophagy regulates intracellular insulin content [53]. These findings support autophagy's protective role against the degeneration of pancreatic β cells and the development of T2DM.

Interestingly, ER which is an integral machinery site for the synthesis of insulin in the pancreatic β cells is highly sensitive to hyperglycaemia‐induced oxidative stress [57]. T2DM‐induced ER stress is resolved by unfolded protein response (UPR). If this process is failed, pancreatic β cells will pass into apoptosis [57]. Activation of UPR triggers the induction of a specific type of autophagy called reticulophagy which removes damaged parts of ER and maintains cellular homeostasis by increasing the expression of Agt8 [58]. Thus, activated autophagy in response to ER stress promotes cell survival [58]. Therefore, autophagy is essential to preserve the functional activity of pancreatic β cells. It has been shown that the autophagy level in pancreatic β cells is positively correlated with insulin a level, indicating that autophagy is linked with insulin storage and release [59]. In addition, defective autophagy promotes the development of mitochondrial dysfunction [59].

Autophagy level in pancreatic β cells is positively correlated with insulin levels, indicating that autophagy is linked with insulin storage and release [59]. Reduction of autophagy function, as evidenced by low Atg7 in mice, decreases pancreatic β cells' functional capacity [51]. Therefore, autophagy is essential for the regulation of the function and integrity of pancreatic β cells.

During IR, insulin secretion is augmented, and the mass of β cells is enlarged to adapt and maintain β cells. In this state, autophagy function is increased to control β cells' dynamic changes. Therefore, failure of autophagy promotes dysfunction of pancreatic β cells, causing the development of T2DM [60]. In the absence of stress, autophagy can act as a housekeeping mechanism, recycling and removing damaged intracellular organelles such as mitochondria. Under stress conditions such as oxidative and ER stress in β‐cells, its disruption results in increased β‐cell stress, cellular degeneration and disruption of insulin secretion [61]. Atg‐7 deficiency decreased β‐cell mass by increasing apoptosis and decreasing proliferation, thereby reducing insulin secretion and inducing glucose intolerance. Likewise, loss of β cell‐specific autophagy increases the accumulation of hIAPP and induces β‐cell apoptosis [61]. However, autophagic flux is not sufficiently activated to compensate for the proteolysis demand in mice with T2DM [60]. Various human studies indicated that pancreatic β cells from T2DM patients have autophagic vacuoles and an increased number of p62 positive β cells [62, 63]. In addition, hyperglycaemia‐induced oxidative stress induces autophagy to protect the retinal cells from the effect of oxidative stress [64].

**FIGURE 3:**
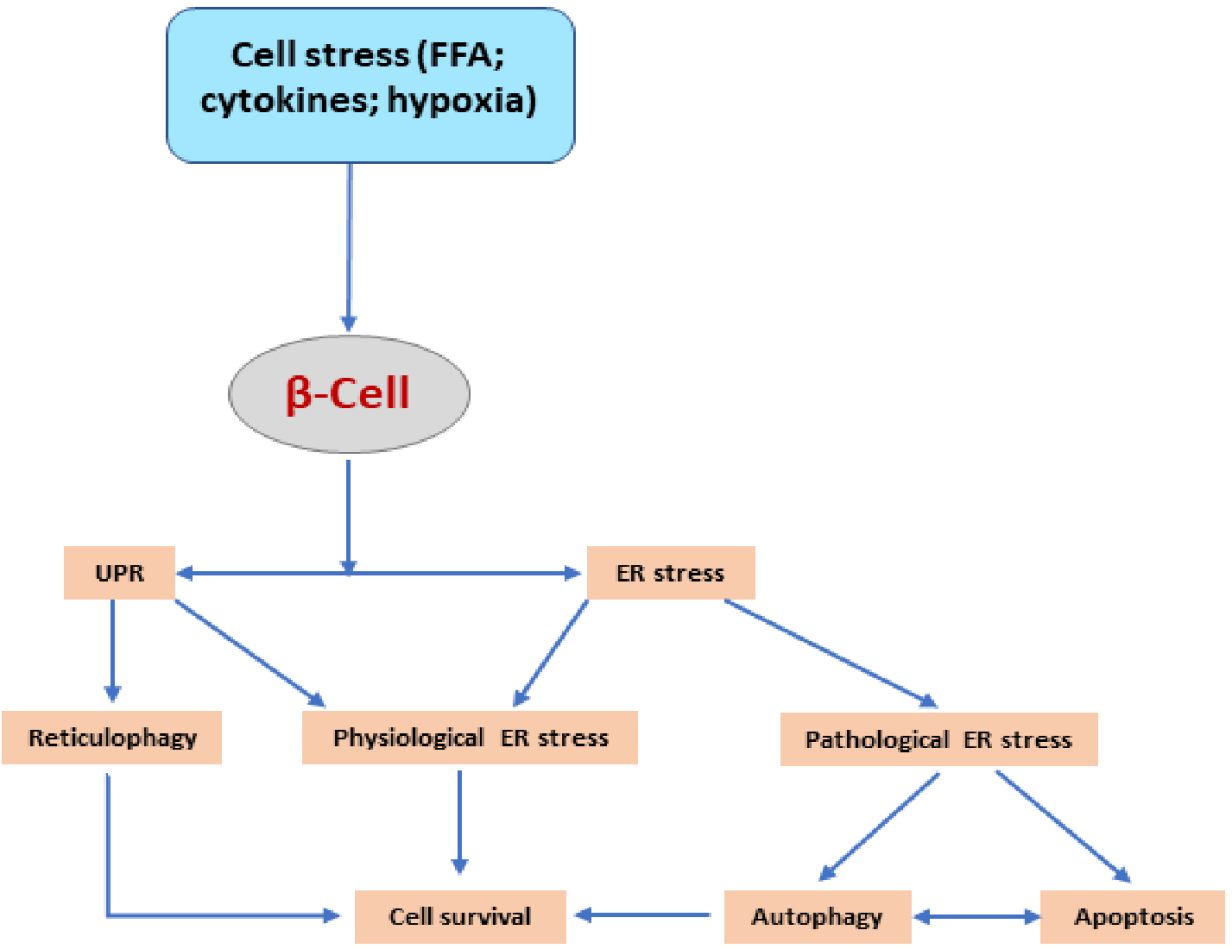
Pancreatic β cells stress and autophagy.

Therefore, activated autophagy protects against ER stress and mitochondrial dysfunction in T2DM (Figure 3).

However, IR and hyperinsulinemia inhibit autophagy and autophagy‐related protein [65]. Insulin can constrain autophagy through activation of mTORC1 leading to the inhibition of ULK1 and inactivation of FOXO transcription factors [61, 66]. Nutrient availability and insulin action activate mTORC1 leading to anabolic responses, though starvation inhibits mTORC1 and thereby activates ULK1, leading to catabolic energy utilisation. Together, FOXO1 integrates signalling from insulin by regulating autophagy gene expression [66]. AKT which regulates mTORC1 and FOXO1/3 represents a key signalling of the intersection between insulin signalling and autophagy. AKT induces the activation of mTORC1 and inhibits FOXO1/3, consequently inhibiting autophagy [67].

Of note, mTOR is regarded as an endogenous inhibitor of autophagy and was reported to increase the progression of diabetic nephropathy [36]. Therefore, a selective inhibitor of mTOR sirolimus attenuates the development of diabetic nephropathy [68]. Thus, sirolimus enhances autophagy [69]. In addition, through induction of autophagy, mTOR inhibitor rapamycin can prevent cardiac dysfunction in T2DM mice [70].

Furthermore, ULK1 and vacuolar protein sorting 34 (Vps34), are concerned with autophagy initiation. ULK1 is activated by AMPK and inhibited by mTOR. ULK1 can regulate different signalling pathways independent of autophagy [71]. Xing et al. demonstrated that upregulation of ULK1 by nitric oxide (NO) can stabilise SIRIT1 independent of autophagy stimulation [72]. ULK1 is reduced in T2DM patients, and the use of the insulin‐sensitising drug metformin has the ability to induce autophagy through the upregulation of ULK1 expression [73].

Defective autophagy delays the removal of aged mitochondria, increasing the progression of oxidative stress and accumulation of misfolded proteins like tau [65]. The expression of LC3 and p62/SQSTM1 was significantly reduced in T2DM patients and was inversely correlated with HbA1c levels, suggesting that the autophagic ability of β‐cells is impaired as the disease progresses [74]. A preclinical study demonstrated that pancreatic β‐cell lines and human islets exposed to high levels of glucose, led to an accumulation of autophagosomes, impaired mitochondria and increased mTOR expression [75]. These findings suggest that high glucose levels block autophagic flux and lead to cell death. However, when treated with rapamycin, the changes in autophagic flux as well as glucose‐induced cell death were reversed [75]. Therefore, defective autophagy in T2DM patients increases the risk for the development of neurodegenerative diseases like Alzheimer's disease (AD) and Parkinson's disease (PD) [76]. Therefore, T2DM, obesity and other cardiometabolic disorders associated with IR are linked with the development of AD [69]. Furthermore, defective autophagy and associated IR promote the development of ER stress, which further induces IR and hyperinsulinemia, causing suppression of autophagy [77].

On the other hand, defective autophagy in the adipose tissue may induce the development of IR. Obesity reduced LC3‐II protein accumulation, indicating attenuated adipocyte autophagic flux, and that these changes were inversely correlated to fat cell size. Further, the obesity‐induced reductions in adipocyte autophagy were reversible and improved after bariatric surgery [78]. In addition, adipocytes isolated from tissue explants of obese individuals have lower autophagic flux than controls [78]. Importantly, adipocytes participate in whole‐body insulin sensitivity through the secretion of adipokines. Thus, the reduction of adipocytes autophagy reduced the circulating levels of the insulin sensitising adipokines such as chemerin and leptin leading to IR and the development of T2DM [79].

Furthermore, Beclin‐1, a positive regulator of autophagy function, is reduced in T2DM patients [80]. A case–control study on 70 T2DM patients and 20 healthy controls revealed that Beclin‐1 serum level was reduced in T2DM patients with renal complications compared to controls [80]. This finding suggests that the reduction of Beclin‐1 could be a possible cause for the reduction of autophagy in T2DM. Zhang et al. illustrated that curcumin modulates the accelerated cell death autophagy and apoptosis in diabetic nephropathy by increasing Beclin‐1 expression in mice [81]. Besides, an herbal medicine, berberine can attenuate the development of diabetic retinopathy through the activation of AMPK, which activates autophagy [82].

These findings suggest that autophagy has a protective role against T2DM (Table 1) by reducing oxidative stress and mitochondrial dysfunction.

**TABLE 1:** The protective role autophagy against T2DM.

Study type	Findings	Ref.
Preclinical	Activation of autophagy attenuates apoptosis of pancreatic β cells induced by lipotoxicity	[16]
Preclinical	Over‐activated of the pancreatic β cells autophagy attenuates FFA‐induced apoptosis	[49]
Preclinical	Autophagy improves pancreatic β cell function and the release of insulin	[50]
Preclinical	Autophagy preserves the homeostasis of pancreatic β cells and prevents oxidative stress‐induced cellular injury	[51]
Preclinical	Autophagy has a protective role against hIAPP‐induced toxicity of pancreatic β cells	[52]
Preclinical	Autophagy regulates the release of insulin from pancreatic β cells	[53]
Preclinical	Reduction of autophagy functions decreases pancreatic β cells' functional capacity	[51]
Preclinical	mTOR inhibitor rapamycin can prevent cardiac dysfunction by activating autophagy in T2DM mice	[70]
Clinical	ULK1 is reduced in T2DM patients, and the use of the insulin‐sensitising drug metformin has the ability to induce autophagy through the upregulation of ULK1 expression	[73]
Clinical	The expression of LC3 and p62/SQSTM1 is significantly reduced in T2DM patients	[74]

### 3.2 Harmful Effects

Different studies revealed that excessive autophagy activation is associated with the induction of autophagic cell death and apoptosis of pancreatic β cells [17, 83, 84]. The role of autophagy in T2DM is believed to be a double sword edge [18]. It has been reported that autophagy‐induced pancreatic β cell death in pancreatic tissues isolated from aged rats. Increased expression of the autophagic markers, Lamp2 and LC3b, was observed with age that correlated with elevation of blood glucose. A significant increase in apoptotic index in older rats indicated that overactivation of pancreatic β cell autophagy is associated with the development of diabetes [84].

In particular, mitochondrial dysfunction and ER stress promote the development of IR [85]. ER‐stress‐induced IR via downregulation expression of insulin receptors and related signalling induces autophagy activation [86]. Furthermore, activated autophagy is linked with the pathogenesis of T2DM [62]. A case–control study on human pancreatic samples from T2DM patients and healthy controls revealed that autophagosomes and autophagic vacuoles are correlated with the level of dead β cells, a biomarker of pancreatic β cells autophagy [62]. Findings from in vitro and in vivo studies revealed that activated autophagy is associated with the loss of pancreatic β cells [87]. Notably, a transcription factor pancreatic duodenal homeobox 1 (Pdx‐1), which regulates the homeostasis of pancreatic β cells, is regarded as an inhibitor of autophagy [88]. Mutation of Pdx‐1 is linked with impairment of glucose tolerance and development of early and late‐onset T2DM by inhibiting a compensatory neogenesis of pancreatic β cells [89]. It has been shown that polymorphism of Pdx‐1 affects insulin secretion in Japanese T2DM patients [90]. Therefore, the Pdx‐1 defect enhances autophagy leading to the progressive loss of pancreatic β cells with the development of T2DM. Furthermore, exaggerated autophagy by hyperglycaemia‐mediated oxidative stress promotes apoptosis of retinal cells with the development of diabetic retinopathy [91].

Furthermore, Beclin‐1, a negative regulator of autophagy function, is reduced in T2DM patients [80]. A case–control study on 70 T2DM patients and 20 healthy controls revealed that Beclin‐1 serum level was reduced in T2DM patients with renal complications compared to controls [80]. This finding suggests that the reduction of Beclin‐1 could be a possible cause for increasing autophagy in T2DM. Zhang et al. [92] illustrated that curcumin modulates the accelerated cell death autophagy and apoptosis in diabetic nephropathy by increasing Beclin‐1 expression in mice. Furthermore, exaggerated autophagy by hyperglycaemia‐mediated oxidative stress promotes apoptosis of retinal cells with the development of diabetic retinopathy [91]. Besides, an herbal medicine, berberine can attenuate the development of diabetic retinopathy through the activation of AMPK, which activates autophagy [82].

Moreover, autophagy‐related genes are augmented and correlated with activated immune cells in pancreatic β cells [93]. Likewise, melatonin attenuates the development of T2DM‐induced osteoporosis by inhibiting osteoblastic autophagy [94]. These observations indicated that activated autophagy is linked with the development and progression of T2DM through modulation apoptosis of pancreatic β cells.

Moreover, activated autophagy attenuates cell survival by inducing the formation of ROS and inducing cell deaths when the apoptotic pathway is compromised [95]. Unchanged gene expression of *Beclin 1* and *Atg1* and reduced transcription of *LAMP‐2* and the genes encoding cathepsin B and D were observed in T2DM islets. The β cell death increased when non‐diabetic islets were exposed to NEFA, and the number of dead β cells with massive vacuole overload was also augmented [96]. NEFA‐exposed cells showed reduced *LAMP‐2* expression. These results validate that autophagy may also contribute to loss of β cell mass [96]. In the later stage of T2DM, sustained metabolic stress can shift autophagy towards promoting β‐cell damage and apoptosis, ultimately resulting in β‐cell failure [97].

These findings highlighted that exaggerated autophagy exerts a detrimental effect on the pathogenesis of T2DM.

Taken together, appropriate autophagy activity is necessary for optimal function of pancreatic β cells. Both defective and exaggerated autophagy of pancreatic β cells is associated with the development of IR and the progression of T2DM and associated complications.

## 4 Conclusion

Basal autophagy is protective by maintaining the homeostasis of cytosolic components. Though induced autophagy in response to starvation, dyslipidaemia and gluco‐lipotoxicity regulates and preserves cellular functions. Autophagy improves the function of pancreatic β cells and the release of insulin. Deregulated autophagy is intricate in the pathogenesis of T2DM. Autophagy preserves the homeostasis of pancreatic β cells and prevents oxidative stress‐induced cellular injury. Thus, pancreatic β cell autophagy seems as an adaptive response to mitigate the development and progression of IR. These findings support the protective role of autophagy against the degeneration of pancreatic β cells and the development of T2DM.

On the other hand, excessive activation of autophagy is associated with the induction of autophagic cell death and apoptosis of pancreatic β cells. Moreover, autophagy‐related genes are augmented and correlated with activated immune cells in pancreatic β cells. Therefore, excessive activation of autophagy plays a detrimental role in T2DM by enhancing apoptosis of pancreatic β cells.

Taken together, the role of autophagy in T2DM is a double sword edge, as basal autophagy protects pancreatic β cells and prevents the development of T2DM. However, over‐activated autophagy is implicated in the apoptosis of pancreatic β cells and the development of T2DM.

## Author Contributions

**Yousef Abud Alanazi:** writing – review and editing (equal). **Haydar M. Al‐kuraishy:** conceptualization (equal), data curation (equal), formal analysis (equal), funding acquisition (equal), investigation (equal), methodology (equal), project administration (equal), resources (equal), software (equal), supervision (equal), validation (equal), visualization (equal), writing – original draft (equal), writing – review and editing (equal). **Ali I. Al‐Gareeb:** conceptualization (equal), data curation (equal), formal analysis (equal), funding acquisition (equal), investigation (equal), methodology (equal), project administration (equal), resources (equal), software (equal), supervision (equal), validation (equal), visualization (equal), writing – original draft (equal), writing – review and editing (equal). **Athanasios Alexiou:** conceptualization (equal), data curation (equal), formal analysis (equal), funding acquisition (equal), investigation (equal), methodology (equal), project administration (equal), resources (equal), software (equal), supervision (equal), validation (equal), visualization (equal), writing – original draft (equal), writing – review and editing (equal). **Marios Papadakis:** conceptualization (equal), data curation (equal), formal analysis (equal), funding acquisition (equal), investigation (equal), methodology (equal), project administration (equal), resources (equal), software (equal), supervision (equal), validation (equal), visualization (equal), writing – original draft (equal), writing – review and editing (equal). **Mostafa M. Bahaa:** conceptualization (equal), data curation (equal), formal analysis (equal), funding acquisition (equal), investigation (equal), methodology (equal), project administration (equal), resources (equal), software (equal), supervision (equal), validation (equal), visualization (equal), writing – original draft (equal), writing – review and editing (equal). **Walaa A. Negm:** conceptualization (equal), data curation (equal), formal analysis (equal), funding acquisition (equal), investigation (equal), methodology (equal), project administration (equal), resources (equal), software (equal), supervision (equal), validation (equal), visualization (equal), writing – original draft (equal), writing – review and editing (equal). **Faisal Holil AlAnazi:** writing – review and editing (equal). **Mohammed Alrouji:** funding acquisition (equal), software (equal), writing – review and editing (equal). **Gaber El‐Saber Batiha:** conceptualization (equal), data curation (equal), formal analysis (equal), funding acquisition (equal), investigation (equal), methodology (equal), project administration (equal), resources (equal), software (equal), supervision (equal), validation (equal), visualization (equal), writing – original draft (equal), writing – review and editing (equal).

## Ethics Statement

The authors have nothing to report.

## Consent

The authors have nothing to report.

## Conflicts of Interest

The authors declare no conflicts of interest.

## Data Availability

All data are available in the manuscript.
